# Presentation, etiology and treatment outcome of neovascular glaucoma in Ekiti state, South Western Nigeria

**DOI:** 10.4314/ahs.v21i3.37

**Published:** 2021-09

**Authors:** Iyiade Ajayi, Olusola Omotoye, Kayode Ajite, Emmanuel Abah

**Affiliations:** Ekiti State University College of Medicine, Ophthalmology

**Keywords:** Neovascular glaucoma, retinal ischemia, rubeosis iridis, secondary glaucoma

## Abstract

**Background:**

Neovascular glaucoma (NVG), a form of secondary glaucoma has varying causes across geographical locations.

**Objective:**

The objective of this study was to determine the presentation, aetiology, and outcome of treatment of patients with NVG in a Nigerian tertiary hospital.

**Method:**

A retrospective review of records of all cases of NVG seen over a 5year period was carried out. Demographic characteristics, presenting visual acuity and coexisting ocular and systemic conditions were noted. Data were analysed with Statistical Package for Social Sciences (SPSS) version 25.

**Results:**

29 eyes of patients with NVG were analysed. Most of the patients (89.70%) presented with visual acuity less than 3/60 in the affected eye. All patients except one were treated with anti-glaucoma medications while only 9(31%) consented to and received anti-vascular endothelial growth factor. No patient had improvement in visual acuity despite resolution of other symptoms at 12week follow up.

**Conclusion:**

NVG though not as common as other forms of glaucoma accounted for a large proportion of monocular blindness in the affected eyes at presentation. There is need for health promotion and education among our people on the need for early preventive eye check practices.

## Introduction

Glaucoma is the leading cause of irreversible blindness globally^1^. It can be primary or secondary. Neovascular glaucoma (NVG) is a form of secondary glaucoma. It usually occurs as a result of ocular or systemic conditions with posterior segment ischemia as a common pathogenesis in most cases^2^–^7^. It is characterized by rubeosis iridis and new vessels on the anterior chamber angle^8^. Presentation varies with the degree of accompanying inflammation and stage of disease. While some may be asymptomatic^9^, others may present with severe painful loss of vision with or without spontaneous hyphaema^7^. The etiology varies in different geographical regions depending on the prevalence of the primary cause of retinal ischemia in the concerned region. In Benin City, Nigeria, Neovascular glaucoma was recorded in 13.6% of patients with retinal vascular occlusion^10^. The hospital prevalence in south-south Nigeria was 0.3%. It accounted for 1.8% and 1% of cases of glaucoma in Southern Uganda and Ghana respectively^1^, ^11^. The literature is replete with different treatment modalities like use of antiglaucoma medications, intravitreal anti-vascular endothelial growth factors like bevacizumab and aflibercept, trabeculectomy with 5 fluorouracil, drainage valve implant, laser cyclophotocoagulation, cyclocryotherapy, panretinal photocoagulation, use of retrobulbar alcohol and evisceration in refractory painful cases.^7^, ^12^–^20^ Reports have shown poor success rates with all these techniques.

## Methodology

Records of all patients with the diagnosis of Neovascular glaucoma (NVG) were retrieved for the period spanning from January 2015 to December 2019. The following information was retrieved from the records: demographic characteristics, presenting visual acuity, location of rubeosis, presence of hyphema at presentation, gonioscopy finding of new vessels on the anterior chamber angle, laterality of NVG, intraocular pressure of the affected eye and the contralateral eye, the number of antiglaucoma medications as well as number of anti-VEGF injections given. Record of hypertension and diabetes, hyperlipidemia and visual acuity at 12 weeks follow up visit was also noted. Etiology of retina ischemia was retrieved from the case record. Data were entered into statistical package for social sciences (SPSS) version 25. Analysis was done for mean and standard deviation for continuous variables while categorical variables were analysed for frequency. Statistical significance was inferred at P less than 0.05. Study was carried out according to Helsinki declaration of 1983.

## Results

A total of 29 patients had diagnosis of NVG during the period reviewed. This constituted 0.05% of the total number of 566 newly diagnosed glaucoma patients seen during the period of study. The ages ranged from 23 to 105 years with a mean age of 62.2 ±20.8. Males accounted for 16 (55.2%) while females accounted for 13 (44.8%). The intraocular pressure (IOP) at presentation ranged from 3 mmHg to 65 mmHg with a mean of 40.7 ±13.3 mmHg. The mean IOP of the contralateral eye was 18.8mmHg ±8.6mmHg. All cases were unilateral with 13(44.8%) involving the right eye while 16(55.2%) affected the left eye. Hyphaema was a finding at presentation in 10(34.5%) patients.

Patients aged 50 years and above constituted 25 (86.2%) of all cases of NVG. Sixteen patients (55.2%) had tertiary education as shown in the table 1.

**Table 1:** Age group of patients with neovascular glaucoma versus level of education Education level Age group in years

	≤40 n(%)	41–49 n(%)	50–59 n(%)	60–69 n(%)	≥70 n(%)
Primary	0	0	2 (6.9)	0	3(10.3)
Secondary	2 (6.9)	0	1 (3.5)	0	1(3.5)
Tertiary	2 (6.9)	0	7 (24.1)	2 (6.9)	5(17.2)
None	0	0	0	0	4 (13.8)
Total	4 (13.8)	0	10 (40.5)	2(6.9)	13 (44.8)

**Figure 1:**
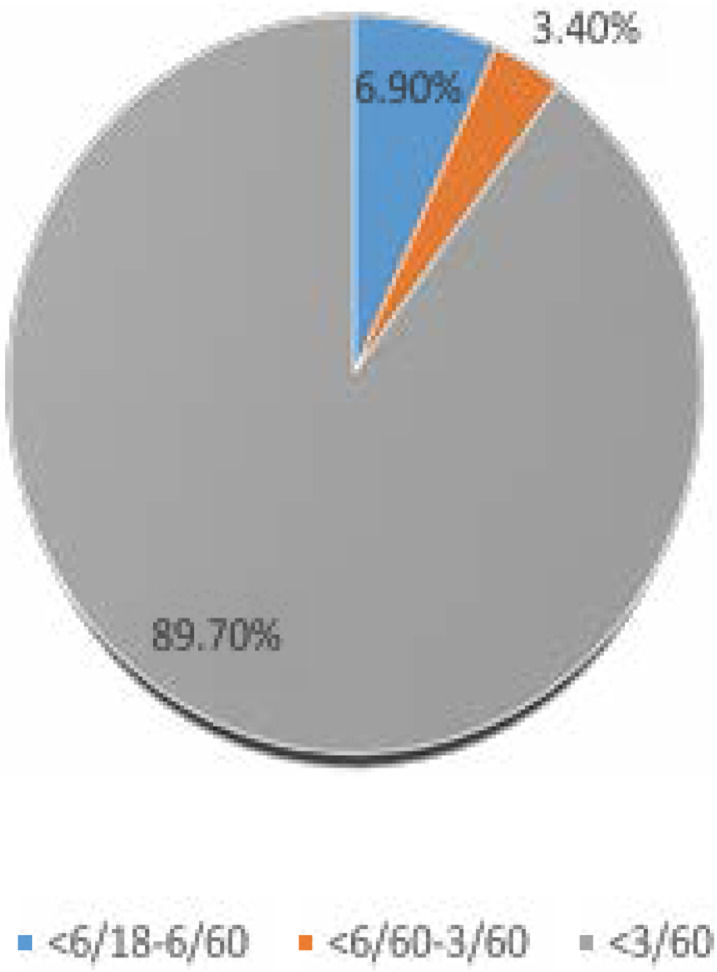
Presenting visual acuity in the affected eye

In the affected eye 89.7% presented with visual acuity less than 3/60 as shown in Figure 1 above.

Six patients (20.7%) had a visual acuity less than 3/60 in the contralateral eye.

Loss of vision, redness and eye ache were the leading presenting complaints among patients. The duration of symptoms ranged from 4 days to 10 years with a mean of 311 days with a mode of 365 days.

Hypertension and Diabetes mellitus were associated systemic comorbidity as shown in Table 4 above. The interval between the diagnosis of Diabetes mellitus and presentation with NVG ranged from 2 to 27years with a mean of 9.0 ±6.9 years while that of hypertension was between 1 and 15 years with a mean of 9.0 years ± 6.8 years. Thirteen patients (44.8%) had both diabetes mellitus and hypertension.

**Table 4:** Occupational distribution of patients with NVG and presenting visual acuity in the affected eye

Occupation	Presenting visual acuity (PVA)	
	>6/60 n (%)	<6/60 n (%)
Blue collar	1(3.4)	13 (44.8)
White collar	1 (3.4)	14 (48.2)

There was no significant difference in the presenting visual acuity among the occupational groups in the patients.

Patients had varying extent of neovascularization on the iris with associated corneal vascularization in 3 (10.3%) patients. One patient (3.4%) had secondary angle closure glaucoma while 28 patients (96.6%) had secondary open angle glaucoma. All patients except one were commenced on anti-glaucoma medications ranging from 1 to 4 classes of drugs. Fourteen patients (48.3%) were on 2 medications, 11(37.9%) patients were on 3 medications while 4 patients (10.3%) were on 4 classes of medications based on the level of their intraocular pressure at the time of presentation. 9 patients (31%) consented to and received anti-Vascular endothelial growth and all had regression of the rubeosis iridis without any visual recovery. One patient was not given any anti-glaucoma medication because of very low pressure due to the presence of retinal detachment on the affected eye. The visual acuity at 12 weeks after presentation were the same as the presenting visual acuity.

**Figure 2:**
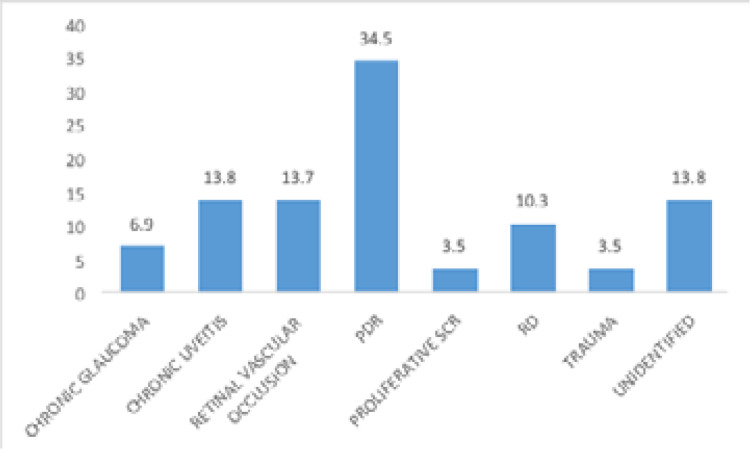
Etiology/identified risk factors in patients with NVG *PDR-Proliferative diabetic retinopathy SCR: sickle cell retinopathy RD: Retinal detachment

The identified leading aetiology were proliferative diabetic retinopathy in 34.5% of patients and chronic uveitis in 13.8% of patients (figure 2). Nine patients (31%) consented to and received anti-Vascular endothelial growth and all had regression of the rubeosis iridis without any visual recovery. Other non-consenting patients were managed with anti-glaucoma medications, anti-inflammatory drugs and mydriatic agent to achieve symptomatic relieve. One patient was not given any anti-glaucoma medication because of very low pressure due to the presence of retinal detachment on the affected eye. The mean intraocular pressure at the last follow up visit was 32.6mmHg ± 21.4mmHg. The visual acuity at 12 weeks after presentation were the same as the presenting visual acuity. Six patients (20.7%) had visual acuity less than 6/60 in the contralateral eye from other eye disorders.

## Discussion

Neovascular glaucoma accounted for 0.05% of all newly diagnosed glaucoma patients seen during the period of study. This is lower than a community based study where it constituted 2% of all glaucoma patients^21^ and 1% of all glaucoma patients in a hospital based study^22^. It accounted for 1.8% and 1% of cases of glaucoma in Southern Uganda and Ghana respectively^1^, ^11^. In this study, the proportion of males was slightly higher than the report from another southwesterNigeria study^22^. A similar gender proportion was reported in a study carried out in south-south Nigeria^23^. We observed a higher mean age of 62.2±20.8 years which is higher than mean of below 60 years in other studies^22^, ^23^ but compares favorably with other reports from Saudi Arabia and China^24^, ^25^. All cases reviewed in this study were unilateral. There were bilateral cases in some other studies from within and outside our nation^22^, ^23^, ^26^. About 1/3rd of our patients had hyphaema at the time of presentation. Spontaneous hyphaema is a known feature of NVG^7^, ^8^, ^27^ and this has been explained to be a result of the friable nature of the immature new vessels that are formed on the iris and anterior chamber angles. The presence of hyphaema may further increase the intraocular pressure in these patients and make them to present with eye pain.

Patients aged 50 years and above constituted 86.2% (Table 1) of all patients with NVG. A possible explanation for this could be the fact that increasing age is a known risk factor for many known ocular disorders that predispose to NVG^23^, ^28^.

Poor vision was a leading feature in most of the patients with NVG in this study with 89.7% presenting with blindness in the affected eye (Figure 1). This could be attributed to the high rate of delayed presentation among the patients as some of them presented as late as 10 years after the onset of symptoms with a mean interval of 31 1days between onset of symptoms and presentation at the eye care centre. A little above half (55.2%) had tertiary education (Table 1) while 13.8% had no formal education. Others had either secondary or primary education. Eye ache, redness photophobia and vision loss were common presenting symptoms in our study (Table 2) and this is similar to findings from other reviews^3^, ^9^, ^23^, ^29^. Spontaneous hyphaema is a documented presenting feature of NVG^30^. Systemic comorbidity observed among the patients were diabetes mellitus and hypertension (Table 3), with 13 patients having both conditions coexisting. This reiterates the risk of ocular ischemia among patients with these conditions. Hypertensive patients are also at risk of carotid occlusive disorder in addition to the fact that both systemic conditions can predispose to vascular occlusive disorders. Systemic conditions like diabetes mellitus and hypertension are well documented predisposing factors to NVG^22^, ^29^, ^31^–^33^

**Table 2:** Presenting complaints among patients

Complaints at presentation	no (%)
Redness	24(82.8)
Vision loss	25(86.2)
Photophobia	10(34.5)
Eye-ache	23(79.3)
Watering	15(51.7)

**Table 3:** Systemic comorbidity among patients with NVG

Systemic comorbidity	no (%)
Hypertension	15 (51.7)
Diabetes mellitus	14(48.3)
Hyperlipidemia	3(10.3)

There was no statistically significant difference in the risk of NVG among our hypertensive and diabetic patients. This also could be alluded to the fact that many of the patients had both conditions coexisting.

The commonest identified etiology identified was proliferative diabetic retinopathy in 34.5% of the patients. (Figure 2) These were jointly followed by chronic uveitis and retinal vascular occlusive disorder in 13.8% each. This agrees with earlier documented reports from other studies^26^, ^29^ Retinal vascular occlusion was the leading etiology in south-south Nigeria^23^ and another study in a southwestern Nigeria community^22^. Other causes include retinal detachment, chronic glaucoma, proliferative sickle cell retinopathy and trauma as shown in figure 2.

There was no difference in the level of presenting visual acuity among the occupational groups whether the white collar or blue collar job. This calls for attention because both groups presented when their visual function had reduced significantly. This implies that there is still a great need for health education in our communities for people to engage in preventive eye checks and present early for eye care as soon as they observe any abnormality in eye function.

All patients who had intravitreal anti-vascular endothelial growth factor had regression of the rubeosis iridis without improvement in vision. Patients however had improvement in visual symptoms like pain and redness at 12 weeks follow up. The delayed presentation observed in the majority of our patients as well as blind status of the eyes at presentation could explain this outcome.

## Limitation

Data collection was done retrospectively. It is possible that some records could have been omitted.

## Conclusion

The proportion of NVG in our centre was low when compared with reports from other studies within and outside the nation. However, the percentage of monocular blindness from this condition was rather high. A great percentage of the patients were above 50 years of age. Proliferative diabetic retinopathy was the leading etiology identified followed by retinal vascular occlusion and chronic uveitis. We also observed late presentation among the patients with no improvement in the level of visual acuity level at 12 weeks follow up visit.

## Recommendation

There is need for more health promotion and education that relates systemic health conditions with visual impairment, with emphasis on the need for early presentation for visual assessments. Furthermore, integrated endocrinology clinics with screening for Diabetic Retinopathy (DR) are essential to identify early complications of DR and prevent visual impairment and Neovascular Glaucoma in this population.
